# 大剂量静脉铁剂与口服铁剂治疗缺铁性贫血疗效及安全性对比：多中心、前瞻性、开放标签随机对照研究

**DOI:** 10.3760/cma.j.cn121090-20240424-00161

**Published:** 2024-12

**Authors:** 倩 张, 虹 王, 彦明 张, 晓莉 李, 耀耀 沈, 楠 魏, 凯 邹, 万秀 苏, 海萍 戴, 德沛 吴, 立民 刘

**Affiliations:** 1 苏州弘慈血液病医院，苏州 215128 Soochow Hopes Hematonosis Hospital, Suzhou 215128, China; 2 苏州大学附属第一医院血液科、江苏省血液研究所、卫生部血栓与止血重点实验室、血液学协同创新中心，苏州 215006 The First Affiliated Hospital of Soochow University, Jiangsu Institute of Hematology, Key Laboratory of Thrombosis and Hemostasis of Ministry of Health, Collaborative Innovation Center of Hematology, Suzhou 215006, China; 3 徐州医科大学附属淮安医院、淮安市第二人民医院血液科，淮安 223300 Huai'an Hospital Affiliated to Xuzhou Medical University, Huai'an Second People's Hospital, Huai'an 223300, China

**Keywords:** 静脉铁剂，大剂量, 口服铁剂, 缺铁性贫血, Intravenous iron, large dose, Oral iron agent, Iron deficiency anemia

## Abstract

**目的:**

比较大剂量静脉铁剂与口服铁剂治疗缺铁性贫血（IDA）的疗效和安全性。

**方法:**

前瞻性随机对照（1∶1）入组2022年6月1日至2024年1月19日苏州大学附属第一医院、苏州弘慈血液病医院及淮安市第二人民医院338例缺铁性贫血患者，其中169例接受大剂量静脉铁剂治疗（静脉组），169例接受口服铁剂治疗（口服组），进行12周观察，主要研究终点为HGB从基线到第4周的变化；次要研究终点为HGB第8、12周的变化，铁代谢参数［血清铁（SI）、转铁蛋白饱和度（TSAT）、总铁结合力（TIBC）、血清铁蛋白（SF）］从基线到第4周的变化及疲劳评分从基线到第4、8周的变化。全程监测两个治疗组的疗效及治疗相关不良反应。

**结果:**

HGB水平在两个治疗组中均得到改善，与口服组相比静脉组HGB早期提升更快。4周时，静脉组HGB由（76.8±15.0）g/L升至（118.0±13.3）g/L，口服组由（77.9±11.6）g/L升至（104.3±15.0）g/L。静脉组较基线升高（40.7±17.3）g/L，显著高于口服组（27.2±17.5）g/L（*P*<0.001）。静脉组比口服组对于改善铁代谢参数的早期效果也更显著，静脉组SI、TSAT、TIBC、SF在4周时较基线改善情况均明显优于口服组（*P*<0.001）。此外，静脉组对于早期改善患者的疲劳评分亦明显优于口服组（*P*<0.001）。静脉组与口服组相比总不良反应发生率相似［3.5％（6/169）对5.9％（10/169），*P*＝0.442］。

**结论:**

大剂量静脉铁剂可以实现HGB早期迅速提升，快速改善SI、TSAT、TBIC、SF及患者疲劳评分，患者耐受性良好。

## Abstract (en)

**Objective:**

This study aimed to compare the efficacy and safety between high-dose intravenous iron and oral iron in treating iron deficiency anemia（IDA）.

**Methods:**

This prospective randomized controlled study（1∶1）enrolled 338 patients with IDA at The First Affiliated Hospital of Soochow University, Suzhou Hongci Hematology Hospital, and Huai'an Second People's Hospital from June 1, 2022, to January 19, 2024. Of all the patients, 169 received high-dose intravenous iron therapy and 169 received oral iron treatment for 12 weeks of observation. Focus on the hemoglobin（HGB）change from baseline to week 4, secondary focus was on the HGB and iron metabolism parameters（serum iron [SI], transferrin saturation [TSAT], total iron binding force [TIBC], serum ferritin [SF]）, and changes in the fatigue score, efficacy, and treatment-related adverse effects were monitored throughout in the two treatment groups.

**Results:**

The HGB levels were improved in both treatments, but the HGB improved faster in the intravenous group compared with the oral group. HGB increased from（76.8±15.0）g/L to（118.0±13.3）g/L in the intravenous group and from（77.9±11.6）g/L to（104.3±15.0）g/L in the oral group after 4 weeks of treatment. The increase from baseline in the intravenous group（40.7±17.3）g/L was significantly higher than that in the oral group（27.2±17.5）g/L（*P*<0.001）. The intravenous group demonstrated a more significant early effect than the oral group in terms of iron metabolism parameter improvement. SI, TSAT, TBIC, and SF increased better from baseline at 4 weeks in the intravenous group than in the oral group（*P*<0.001）. Additionally, the intravenous group exhibited better fatigue scores for early improvement than the oral group（*P*<0.001）. The incidence of total adverse effects was similar in the intravenous group as compared to the oral group（3.5％ [6/169] *vs* 5.9％ [10/169], *P*＝0.442）.

**Conclusion:**

High doses of intravenous iron quickly boost HGB early, causing rapid improvement in SI, TSAT, TBIC, SF, and patient fatigue scores. The patient was well tolerated.

缺铁性贫血（Iron deficiency anemia, IDA）是最常见的贫血性疾病，根据2021年WHO报告，全球有高达23.6亿的贫血患者，其最重要的原因是缺铁[1]。IDA严重影响患者生活质量，去除病因和补充铁剂是IDA的主要治疗手段。目前临床上常用的补铁药物主要有口服铁剂和静脉铁剂，口服铁剂应用早、使用方便，但由于其吸收有限、治疗周期长且胃肠道等不良反应发生率高，患者依从性较差，使得相当比例患者的补铁量不能达到要求。在需要快速纠正贫血的情况下，口服铁剂往往不能满足临床需要[2]–[3]。对于口服铁剂不耐受或疗效不佳的IDA患者，应考虑静脉铁剂治疗[4]。静脉铁剂比口服铁剂可以更快更多提供铁供应，且可以避免胃肠道不良反应。但输注铁剂可能会出现输注过敏反应、药物外漏致局部组织损伤、低血压等不良事件。近年来，可以实现单次大剂量输注的新一代静脉铁剂异麦芽糖酐铁已在国内上市[3],[5]–[6]。因其在国内上市时间相对较短，目前尚缺乏大宗针对中国人群用药的相关数据。鉴于以上背景，本研究我们采用多中心、前瞻性、随机对照的方法，对大剂量静脉铁剂及口服铁剂治疗IDA患者的疗效及安全性进行对比分析。

## 病例与方法

1. 研究设计：本研究为多中心、前瞻性、开放标签随机对照研究，旨在比较静脉铁剂异麦芽糖酐铁与口服铁治疗IDA的疗效和安全性。按照1∶1随机入组，比较大剂量静脉铁剂及口服铁剂在中国IDA人群中的有效性、安全性、起效速度、FACIT-F评分恢复速度。主要研究终点为治疗4周时HGB水平，次要研究终点为治疗8周及12周时HGB水平、治疗第4周时铁代谢参数［血清铁（SI）、转铁蛋白饱和度（TSAT）、总铁结合力（TIBC）、血清铁蛋白（SF）］及治疗第4、8周时疲劳评分变化。采用完全随机对照设计的两样本均数计算样本量，设*α*＝0.05，*β*＝0.10，根据相关文献确定*µ*_1_−*µ*_2_＝4，*σ*²＝10²＝100，计算得出*n*1＝*n*2＝132，考虑到病例脱落因素，增加15％的样本量，所以各组计划纳入样本量增加至156例。

2. 研究对象：入组标准：年龄≥18岁、不同病因导致的IDA患者，SF<100 µg/L，TSAT<20％。排除标准：①怀孕、哺乳或计划怀孕的妇女；②已知对其他肠外铁剂发生过严重超敏反应患者；③非IDA（如溶血性贫血）患者；④失代偿性肝硬化或活动性肝炎（丙氨酸转氨酶>正常值上限3倍）患者；⑤有2种以上药物过敏史的患者。2022年6月1日至2024年1月19日在苏州大学附属第一医院、苏州弘慈血液病医院及淮安市第二人民医院分别对符合纳入标准的IDA患者进行招募，IDA的诊断标准符合《血液病诊断及疗效标准》[7]。除外肿瘤、风湿及免疫系统疾病，排除先天性及其他获得性骨髓造血衰竭性疾病。

3. 治疗方法：静脉组应用异麦芽糖酐铁治疗，按照简化表来确定铁需求，推荐剂量如下：①体重<50 kg的患者给予500 mg；②HGB≥100 g/L时，体重50～70 kg的患者给予1 000 mg，体重>70 kg的患者给予1 500 mg；③HGB<100 g/L时剂量增加，体重50～70 kg的患者给予1 500 mg，体重>70 kg的患者给予2 000 mg[8]，每周总剂量不应超过20 mg/kg，如果大于20 mg/kg，则分两次给药，间隔1周。单次剂量≤1 000 mg至少静脉滴注15 min，单次剂量>1 000 mg至少静脉滴注30 min。口服组应用多糖铁复合物300 mg/d，待HGB恢复正常后继续使用4个月。

4. 疗效及安全性评价：检测治疗前基线及治疗4、8、12周时HGB水平，治疗4周时SI、TSAT、TIBC、SF水平。评估药物临床疗效同时全程监测治疗过程中安全性，FACIT疲劳量表评估患者治疗前及治疗第4、8周时FACIT-F评分。全程注意患者不良反应情况并及时采取相应处理措施。

5. 统计学处理：使用SPSS 20.0软件进行统计分析，定量数据以均数±标准差表示（符合正态分布），分类资料采用百分比表示。定量数据应用独立样本*t*检验进行分析，定性资料采用卡方检验或Fisher精确概率法进行分析。*P*<0.05为差异有统计学意义。

## 结果

一、基线特征

共入组370例IDA患者，有32例入组后退组。其中静脉组和口服组各16例。口服组退组的原因包括：8例患者无法耐受口服铁剂不良反应退组，改用其他铁剂治疗；3例患者口服2周铁剂后自行停药退组；3例患者口服铁剂3周后门诊失访退组；2例患者不配合定期监测HGB及铁代谢参数等指标退组。静脉组退组的原因包括：7例患者治疗后门诊失访退组；2例患者不能耐受静脉铁剂不良反应退组；4例患者静脉补铁后自行口服补铁退组；3例患者静脉补铁后数据不全退组。

最终纳入分析的口服组169例，静脉组169例。静脉铁剂使用剂量：500 mg 1例（0.6％）、1 000 mg 85例（50.3％）、1 500 mg 52例（30.8％）、2 000 mg 30例（17.8％）、2 500 mg 1例（0.6％）。两组患者一般资料如表1所示，其中性别、年龄、治疗前HGB、SI、SF及FACIT-F评分差异均无统计学意义（均*P*>0.05），静脉组TIBC高于口服组（*P*＝0.032），静脉组TSAT低于口服组（*P*＝0.026）。

**表1:** 缺铁性贫血患者接受大剂量静脉铁剂或口服铁剂治疗两组一般临床特征比较

临床特征	口服组（169例）	静脉组（169例）	*P*值
性别［例（％）］			1.000
女	162（95.9）	162（95.9）	
男	7（4.1）	7（4.1）	
年龄（岁，*x*±*s*）	41±12	41±11	0.500
血红蛋白（g/L，*x*±*s*）	77.9±11.6	76.8±15.0	0.400
血清铁（µmol/L，*x*±*s*）	5.1±8.3	3.9±3.7	0.120
总铁结合力（mol/L，*x*±*s*）	85.7±16.5	90.6±19.2	0.032
转铁蛋白饱和度（％，*x*±*s*）	7.9±16.6	5.3±9.5	0.026
血清铁蛋白（µg/L，*x*±*s*）	9.6±9.5	10.7±14.0	0.500
FACIT-F评分（分，*x*±*s*）	22±5	23±6	0.200

二、疗效

经治疗后两组患者的HGB水平均有改善，且在每个监测点都表现为持续、显著的升高，但静脉组HGB变化在每个节点始终优于口服组，尤其在第4周时优势最明显：静脉组HGB由（76.8±15.0）g/L升至（118.0±13.3）g/L，口服组由（77.9±11.6）g/L升至（104.3±15.0）g/L，静脉组HGB较基线升高（40.7±17.3）g/L，显著高于口服组的（27.2±17.5）g/L（*P*<0.001）。

随访至8周时，两组HGB上升平均速率均减慢，静脉组HGB升至（122.9±7.9）g/L，口服组HGB升至（113.1±17.4）g/L（*P*＝0.033），静脉组与口服组HGB水平较基线升高幅度差值达最大17.4 g/L［（52.4±14.8）g/L对（35.0±18.2）g/L，*P*<0.001］；继续观察至12周时，两治疗组HGB水平趋于稳定，静脉组达到（126.2±23.1）g/L，口服组达到（114.5±13.9）g/L（*P*＝0.025），两组较基线升高差值缩小至9.6 g/L（*P*＝0.082）。

单因素分析表明，采用静脉铁剂治疗可以使患者HGB快速上升，4周、8周时静脉组与口服组患者HGB水平较基线升高幅度分别为（40.7±17.3）g/L对（27.2±17.5）g/L（*P*<0.001）、（52.4±14.8）g/L对（35.0±18.2）g/L（*P*<0.001）。纳入性别、年龄及治疗方式后进行多因素分析显示，采用静脉铁剂治疗是HGB快速上升的唯一因素（*P*<0.05）。

比较两组患者治疗第4周时铁代谢参数改善情况。在增加储存铁、改善铁代谢方面，静脉组优于口服组，SI、TSAT、TIBC、SF在4周时静脉组较基线改善情况均优于口服组（*P*<0.001），其中SF在4周、8周时均高于口服组，4周时两组SF差距更显著［（107.6±81.3）µg/L对（12.6±17.3）µg/L，两组差值95 µg/L，*P*<0.001］。

根据FACIT-F评分，结果显示随着治疗进行两组患者疲劳评分在各评估时间点均有改善。治疗4周时静脉组患者FACIT-F评分改善优于口服组（*P*<0.001）；至8周时静脉组FACIT-F评分进一步改善，但口服组8周时FACIT-F评分较4周时改善不明显［4周时，静脉组由（23.2±5.6）分升至（45.1±2.2）分，*P*<0.001，较基线升高（21.6±4.6）分；口服组由（22.3±5.0）分升至（38.6±4.9）分（*P*<0.001），较基线升高（17.1±6.6）分；8周时，静脉组FACIT-F评分继续升至（49.1±1.1）分（*P*<0.001），较基线升高（27.5±5.0）分；口服组升至（41.2±5.7）分（*P*<0.001），较基线升高（18.5±5.2）分］。

三、治疗相关不良反应

静脉组与口服组相比总不良反应发生率差异无统计学意义［3.5％（6/169）对5.9％（10/169），*P*＝0.442］。口服组有8例患者治疗期间出现恶心、便秘等消化道症状，其中包含1例患者合并腹泻、情绪急躁，1例患者诉用药期间乏力感。静脉组有2例输注过程中出现低热，2例用药后出现延迟孤立反应，1例输注后出现血管性水肿，1例输注过程中出现短暂血压下降、胸闷，给予暂停输注，应用抗过敏药物后缓解，后缓慢顺利输注。其中无血栓和栓塞事件发生，无肝功能及肾功能受损情况，无心血管事件，无其他明显不良反应的发生。总体来讲，两组患者不良反应轻微，给予对症处理后均好转。

## 讨论

贫血是威胁人类健康的重大问题，据统计全球27％的人口患有贫血[9]，超过60％的贫血主要原因被确定为IDA[10]，我国仅有不到20％贫血患者接受治疗，即使是重度贫血也只有50％患者接受治疗[11]。贫血发病率高，但重视度不够是我国面临的主要困难。

IDA严重影响患者的生活质量，主要治疗手段是去除病因和补充铁剂。IDA的治疗药物主要为口服和静脉铁剂，口服铁剂虽使用方便，但仍存在治疗周期长[12]–[13]、胃肠道不良反应较明显[3]、患者依从性差、补铁不足的问题。有研究结果显示，口服铁剂可能增加患肠炎的风险[3],[14]。因此，口服铁剂并不适用于所有贫血患者。

静脉注射铁剂代替口服铁剂治疗IDA已经过多项研究证实[15]–[17]，目前关于大剂量静脉铁剂与口服铁剂治疗IDA疗效及安全性对比尚缺乏前瞻性的临床研究，我们通过多中心、前瞻性、开放标签对照研究证实大剂量静脉铁剂可以快速恢复IDA患者的HGB和储备铁，明显改善IDA患者的FACIT-F评分。在本研究人群中，各治疗组之间HGB水平的增加具有可比性，且在第4周时静脉组比口服组显著提高HGB水平（*P*<0.001）。在改善铁代谢、补充储存铁方面静脉组较口服组早期效果也更显著，SI、TSAT、TIBC、SF在4周时较基线改善幅度优于口服组（*P*<0.001），此外，静脉组早期显著改善患者的FACIT-F评分（*P*<0.001），大剂量静脉补铁不良反应发生率低（3.5％），患者耐受性良好。口服铁生物利用度低，不足10％，对于已经存在消化系统疾病、心力衰竭、慢性肾脏病等基础疾病的IDA患者往往同时存在胃肠吸收障碍，对于短期需要迅速纠正贫血，比如近期要做手术的患者，更适合选择大剂量静脉铁剂以快速提升HGB及铁代谢指标[14],[18]。通过FACIT疲劳量表[19]，我们发现相较于口服组，大剂量静脉铁剂组FACIT-F评分显著升高，这意味着大剂量铁剂可显著减低患者疲劳程度。疲劳是导致慢性贫血患者生活质量下降的主要原因之一。较高的FACIT-F评分意味着疲劳程度降低，患者能够更好地参与日常活动，提高生活质量。当患者感受到治疗对疲劳的显著改善时，他们可能会更愿意坚持治疗，从而进一步改善贫血的其他症状，是慢性贫血管理成功与否的重要指标。

一项由200余家医疗机构参与，总计纳入7 642例IDA住院治疗的患者关于静脉用蔗糖铁治疗IDA现状的真实世界研究表明：大多数患者庶糖铁治疗剂量不足，蔗糖铁使用总剂量平均值仅为510 mg，达到或超过1 000 mg的占12.2％。蔗糖铁治疗后HGB升高≥20 g/L的患者比例不到20％，当蔗糖铁使用量达到1 000 mg及以上时，这一比例显著提升[20]。蔗糖铁可能因住院时间长或需频繁就医等原因影响患者依从性，导致使用剂量不足，从而未达到治疗效果。经计算，不同病因的IDA患者的需铁量均>1 000 mg[21]。异麦芽糖酐铁由于其创新的矩阵式结构和低免疫原性的特点[22]，可以实现每次1 000 mg以上的输注。临床研究显示，给药后2～4周可达满意疗效，而4～8周之后疗效保持[23]–[24]。IDA大部分受其原发病影响，治疗周期相对较长，即便是既往使用静脉铁剂仍然因需要反复多次输注，临床实践中依从性差，导致大多数患者治疗剂量不足。当前，补铁剂量不足是IDA治疗中存在的最大问题，大剂量静脉铁剂相较于传统静脉蔗糖铁，可以减少用药频率，从而减少患者往返医院次数、减轻治疗痛苦及焦虑，有利于增强患者治疗依从性，提升治疗效果[18],[25]。

我们对使用静脉铁剂的主要顾虑是可能产生的不良反应，如过敏反应、铁过载而致严重细菌感染的风险增加、药物外漏、低血压、肝损伤等[26]。我们纳入的169例患者应用异麦芽糖酐铁后6例出现输液反应，包括低热、肌肉痛、孤立反应、局部血管性水肿及短暂血压下降、胸闷，均为自限性不良反应，总体发生率低，无消化道不良反应、无血栓和栓塞事件发生，无肝功能及肾功能受损情况，无心血管事件，无其他明显不良反应的发生，患者对其耐受性良好，这可能与异麦芽糖酐铁的代谢途径有关[27]。

综上所述，大剂量静脉铁剂的长期疗效与一线口服铁剂相当，患者耐受性良好，且治疗早期比口服铁剂提升HGB更迅速，可快速纠正贫血，改善患者疲劳状态，单次可输注≥1 000 mg，1～2次即可补足储备铁。因此，对于口服铁剂治疗反应差、不耐受、依从性差，或短期需快速纠正贫血的IDA患者，本研究提示采用大剂量静脉铁剂也是有效且安全的治疗选择。
